# An Appraisal of Anatomical and Limited Hepatectomy for Regional Hepatolithiasis

**DOI:** 10.1155/2010/791625

**Published:** 2010-03-15

**Authors:** Hui Jiang, Hong Wu, Ying-long Xu, Jing-zhou Wang, Yong Zeng

**Affiliations:** Department of Hepatobiliary Pancreatic Surgery, West China Hospital, Sichuan University, Chengdu, Sichuan 610041, China

## Abstract

*Aim*. Determination of first line treatment with limited hepatectomy or Anatomical hepatectomy provides better clinical outcome. *Methods*. Immediate and long-term outcomes of 106 patients who underwent partial hepatectomy for RH at our institution from January 2001 to February 2005 were analyzed retrospectively. Clinical end-points included time to recovery of hepatic function, residual stones, infection of the liver remnant, bile leakage, recurrent stones, morbidity, and mortality. *Results*. LH was performed in 59 patients and AH in 47 patients as first-line treatment. The time of hepatic function recovery was not statistically different between the two groups (*P* > .05). However, Patients in AH group suffered from less residual stones (*P* < .05), less infection of the raw surface of liver remnant (*P* < .05), and less bile leakage (*P* < .05), with a median follow-up of 40.3 ± 0.8 months (range 3–48), and AH group suffered a less recurrent stone rate (*P* < .05). No difference in morbidity, and mortality rates between the two groups. *Conclusion*. AH is a safe and effective treatment for RH, with a fair rate of surgical complications, it should be considered as first-line treatment of RH.

## 1. Introduction

Hepatolithiasis, which is defined as the occurrence of stones in any intrahepatic bile duct proximal to the confluence of the right and left hepatic ducts, is prevalent disease in Southeast Asia and is especially prevalent in China [1, 2]. Su et al. [3] reported that the relative incidence to be 20% of all cases of gallstone disease. Huang ZQ reported hepatolithiasis is now become to be mild symptoms, regional types, and early disease courses [4]. To prevent immediate and late sequelae of hepatolithiasis, such as suppurative cholangitis, septicemia, secondary biliary cirrhosis with resultant portal hypertension, bleeding varices, and hepatic failure [5], aggressive treatment is needed. Surgeons have persistently explored for its treatment, from bile duct exploration to liver parenchyma incision, cholangioscopic lithotomy, and liver resection; all are for one single purpose: “to remove lesions, extract up stone, correct stricture, keep drainage thoroughly, and prevent recurrence”. Hepatectomy seems to be the most definitive approach for hepatolithiasis, because it can remove the stones and the biliary stricture simultaneously, thus reducing the risk of recurrent stones [6–9]. Hepatectomy offers the optimal treatment for this disease in selected patients [8–11]. Unfortunately, immediate and long clinical outcomes after partial hepatectomy is still unsatisfactory because of the high incidence of complications such as bile leakage, hepatic section infection, subdiaphragmatic hydrops infection, and residual and recurrence of stones [11–13].

Because the majority of patients with hepatolithiasis have underlying cirrhosis, preservation of enough liver parenchyma is critical for the success of the operation [14]. Many surgeons preferred LH for hepatolithiasis and just extract up stones with other methods such as combined cholangioscopic lithotomy intra- and postoperation.

To date, no comparative study or randomized trial has addressed the issue of managing RH with LH and AH as first-line treatment to compare their relative efficacies. At our institution, all hepatolithiasis are referred to the surgical unit. We hypothesize that AH can produce better clinical outcome than LH for RH.

## 2. Patients and Methods

### 2.1. Patients

A search of the departmental database was carried out for patients who underwent treatment of hepatolithiasis. The 4-year period of January 2001 till February 2005 inclusive was reviewed. Regional hepatolithiasis was defined as stones locally distributed in one or several hepatic segments along the intrahepatic biliary tree, often complicated with hepatic duct stenosis in the affected area, as well as atrophy of involved hepatic segments. Patients were included in the study if they met the stone-grouping criteria described in the 2007 Guidance for Diagnosis and Treatment of Hepatolithiasis, issued by the Biliary Surgery Group of the Surgery Branch of the Chinese Medical Association. Patients were excluded if they were not amenable to hepatectomy, or if there was a concomitant pathology that required urgent surgical intervention. These included patients with acute cholecystitis, liver abscess, and acute cholangitis from choledocholithiasis, those who complicated cholangiocarcinoma confirmed preoperative were also excluded. Patients with hepatolithiasis suffered both liver resection and cholangiojejunostomy that were excluded as well.

In all patients, the clinical parameters, hematologic and biochemical findings, and radiologic findings were obtained from the case records. The extent and severity of the disease were evaluated by biochemical tests, instrumental examination including hepatic ultrasonography, abdominal spiral CT-scan, magnetic resonance cholangiopancreatography (MRCP), and, in some cases, endoscopic retrograde cholangiopancreatography (ERCP) and percutaneous transhepatic cholangiography (PTC).

The approach to hepatectomy of RH was determined and carried out by the individual surgeons. The unique situation in our department was that 1 team of surgeons favored the use of LH and 1 favored AH of RH for first-line treatment. Their choice of treatment was consistent over the period of the study, and this allowed for comparison of treatment between the 2 groups.

### 2.2. Surgical Technique

 The indications for hepatectomy for these patients were intrahepatic lithiasis that caused liver fibrosis or atrophy or fibrotic stricture of left or right hepatic duct or their second/third branches.

Anatomic hepatectomy, in the form of segmentectomy and/or subsegmentectomy as described by Makuuchi et al. [15], was our preferred surgical method in AH group. Operative procedures in anatomical group were defined following the terminology proposed by Strasberg [16]: segmentectomy (resection of Cournand's segment) [15, 17], sectionectomy (resection of Healey's segment) [18], hemihepatectomy, or trisectionectomy. In segmentectomy, the hepatic parenchyma was transected at the intersegmental plane as described by Cournand. If the hepatic parenchymal transection plane needed to go beyond the intersegmental plane to achieve the desired extent of resection margin, the small portal branches supplying the liver parenchyma up to the aimed transection plane were punctured under ultrasound guidance, and then liver subsegmentectomy was performed either alone or in combination with segmentectomy along the plane of demarcation as delineated by intraoperative ultrasound. We always resected the whole liver segment(s) which contained the expanded and strictured duct in anatomical resection, as well as the drainage area of bile duct.

Limited hepatectomy, in the form of nonanatomic resection or wedge resection, was performed with no regard to segmental or subsegmental plane. In LH group, only liver fibrosis or atrophy tissue was resected and then extracted stones with stone forceps or cholangioscopy from the expanded bile duct of the raw transection surface. Wedge resection was only performed for the superficial Calculi situated at the border of more than one liver segment.

In both groups, a T-tube was routinely inserted after hepatectomy for postoperative cholangiography and choledochoscopy via the T-tube route. After liver resection, intraoperative choledochoscopy was routinely used instead of intraoperative cholangiography for confirmation of stone clearance. Hepatic inflow occlusion to control blood loss during liver parenchyma transection was achieved by clamping the portal triad structures (hepatic artery, portal vein and common bile duct) with a tourniquet if necessary. Drains were placed routinely in the subphrenic space or the Winslow's foramen for draining peritoneal fluid in all patients. Drains were removed when the drainage became serous in nature and not bile-stained or blood-stained at postoperative day 3 to 6.

### 2.3. Postoperative Treatment

The postoperative treatment included the return to oral feeding after 48 hours via nasogastric aspiration, inhibitors of gastric acid secretion and broad-spectrum antibiotics, daily biochemical monitoring of blood crasis, and of hepatic function for the first 5 postoperative days.

### 2.4. Follow-Up

After discharge, the clinical conditions as well as the hepatic function of all patients were monitored according to a median follow-up of 40.3 ± 0.8 months. Liver morphological findings were obtained by ultrasonography and (or) abdominal CT.

The clinical end points used in the study include the following.

- Time of hepatic function recovery: the time was defined as the number of days from the day of initiation of hepatectomy to the time that the biochemical indicator had declined to normal according to daily hepatic function of the first five postoperative days.
- A residual stone was defined as Calculi in the intrahepatic duct within 3 months after hepatectomy; diagnosis is frequently with cholangiography, sonography, or abdominal CT.
- Infection of liver remnant was defined as the remnant surface had necrosis and liquation and had the evidenced of infection from 1 week to 1 month: (1) WBC counts increase beyond 10 × 10^9^/L or neutrocyte exceed 80%; (2) .liquor pruis fluid in peritoneal drainage tube, had a fever of 37.5°C or more for two consecutive days.
- Postoperative bile leakage was diagnosed based on the following criteria: (1) bile fluid in the peritoneal drainage or oozing from the wound and (2) evidence of bile leakage proven by cholangiography through T tube.
- Recurrent stones are defined as Calculi in the intrahepatic duct within a median follow-up of 40.3 ± 0.8 months after hepatectomy; diagnosis is frequently with cholangiography, sonography, or abdominal CT.
- Mortality was defined as death within 30 days or within the same hospital admission.

### 2.5. Analysis and Statistics

Patients' demographic data and clinical outcomes were collected retrospectively. The database was established in SPSS13.0 software. All continuous data were expressed as median ± SEM. Student *t* test was used to compare continuous data, and chi-square test or the Fisher exact test was used to compare discrete data. *P* value less than  .05 was considered statistically significant.

## 3. Result

A total of 120 patients were treated with LH and AH over the 4-year period of the study. After applying the inclusion and exclusion criteria of the study, 106 patients were eligible for further analysis. Fourteen patients were excluded; 1 had suspected rupture of abscess, 7 had concomitant acute cholangitis and 5 acute cholecystitis requiring urgent surgical intervention, and 1 had suspected cholangiocarcinoma. There were a total of 41 men and 65 women, with a median age of 52 ± 1.2 years (range: 24–84). All patients were ethnic Chinese. Fifty-nine patients underwent LH, and the other 47 underwent AH as first-line treatment. The clinical and laboratory parameters of both patient groups are compared in Table 1. There was no significant difference in the patient demographics, comorbidities, and laboratory parameters between the 2 treatment arms.

The characteristics of the hepatolithiasis treated in both groups are shown in Table 2. All cases were regional type, and the distributions of stones in both groups are similar. The peak incidence of hepatolithiasis in our cohort occurred in left lobe (76/106). 

Operative procedures in anatomical group were one or two segmentectomy in 17 cases, sectionectomy in 25 cases, hemihepatectomy in 4 cases, and trisectionectomy in 1 case (Table 3). Mean operation time was 284 ± 11 minutes in anatomical group and 259 ± 18 minutes in nonanatomical group (*P* > .05). Mean operative blood loss was 783 ± 62 mL and 657 ± 103 mL, respectively (*P* > .05).

There were no death in both LH and AH group. The clinical outcome in both treatment arms is shown in Table 4. There was no significant difference between LH and AH in time to restoration of liver function when treatment was successful. However, the immediate stone clearance rate was better in AH group (45/47); only 2 patients had residual stones. After subsequent choledochoscopic lithotripsy by T-tube route, both patients got a final stone clearance. 1 patient with infection of the liver remnant achieved recovery through percutaneous drainage guided by B-us and broad-spectrum antibiotics. There was no occurrence of bile leakage in AH group. And during a median follow-up, no recurrent stones developed. Our series showed that the morbidity of AH was 6.4% (3/47), compared with 47.5% (28/59) in LH. 

 However, LH group suffered a less immediate stone clearance rate (46/59), 9 patients got a final stone clearance after choledochoscopic lithotripsy by T-tube route, and 2 patients had a second procedure of bile duct exploration because of accompaniment bile leakage. The other 2 patients with no symptom give up further therapy. More infection of liver remnant (21/59) and bile leakage (7/59) occurred in LH group; those patients suffered a long-time drainage and hospital stay. During a median follow-up of 40.3 ± 0.8 months, 12 of the 59 patients had recurrent stones development, confirmed by CT scan and cholangiography.

## 4. Discussion

As a common disease of the biliary system, hepatolithiasis is of complicated pathological changes with high residual stone rate, recurrence rate, and resurgery rate; also it is a significant cause for deaths due to benign biliary disease in our country. The treatment is mainly dependent on surgical operation, in accordance with the principle of “removing lesions, extracting up stone, correcting stricture, maintaining drainage thoroughly, and preventing recurrence”. The principles of definitive surgery for hepatolithiasis comprises complete removal of intrahepatic and extrahepatic stones, strictured duct, and the establishment of satisfactory drainage of the affected segments of biliary tree [19]. The surgical methods include intrahepatic bile duct exploration, partial hepatectomy, hilar biliary stricture reconstruction, and liver transplantation [20]. In recent years, liver resection has been applied in patients with hepatolithiasis more and more extensively [21–23]. Nevertheless, the previous understanding of “removing lesions” was limited to removing stone, biliary stricture, and hepatic abscess, and so forth and overlooked the fact that affected liver lobes (segments) and biliary tree are all lesions. 

Some surgeons preferred LH for hepatolithiasis for the reason of preserving enough functioning liver parenchyma to allow the patient to survive the operation, and preservation of enough liver parenchyma is critical for the success of the operation [14]. And resection of atrophic segments was not technically difficult, as most of the tissue had been destroyed. Stone removal could also be facilitated through the transected duct orifices. It seems a safe procedure with a scare possibility of liver failure. However, AH is unlikely to be ethically acceptable because surgical stress of anatomical major resection is apparently greater and many surgeons will be reluctant to choose possibly unnecessary major hepatectomy for regional hepatolithiasis. And many other methods such as cholangioscopic lithotripsy can also be helped to clean stones intra- and postoperation. To minimize total surgical stress, limited small resection was preferentially applied in regional hepatolithiasis. Lithotripsy is effective in removing stones, but residual stones are common after either intra- or postoperative lithotripsy, especially when a segment of liver is packed with stones. 

LH targeted at removal of the destroyed lobe and preservation of good liver tissue should be the goal. This was shown in our series to have achieved bad outcomes. Liver tissues around bile duct with chronic inflammation were often maintained in LH. It was found through subsequent clinical observation that, although such hepatic tissues possibly maintained hepatic cell function, long-standing chronic inflammation of hepatic tissues around bile duct, as well as the unavoidable residual bile sands, would be mostly the source for later cholangitis and recurrent stone, and hepatic cell function would be lost eventually. Especially in patients with cholangitis, the intrahepatic bile duct was full of purulent floccules, as well as inflammatory bile thrombi. It is difficult to eliminate such mixtures by the bile duct itself, which is the major factor for persistent fever and recurrent stones postoperatively. Therefore, “removing lesions” plays a crucial role in treatment of hepatolithiasis. The lesions of hepatolithiasis include stones, stricture, dilation, and also affected hepatic tissues. According to pathological features, the diseased scope of RH is strictly distributed along the biliary tree, which included stone-bearing segments that are not atrophic.

A strict, hepatic segment-based regular resection for regional hepatolithiasis is needed, and patients with RH would definitely benefit from a more aggressive approach. AH is the optimized method for this condition, which completely removes diseased bile duct and its drainage area, thus reducing the risk of long-term recurrence of stones and may also prevent cholangiocarcinoma possibly complicated in hepatolithiasis [9]. Our experience was that, guided by intraoperative ultrasound, a segment or subsegment-based liver resection was carried out strictly. After liver resection and hemostasis was achieved, bioglue was used for sealing the raw surface of liver remnant. As is shown in Table 4, the results of AH group were good when contrasted with those of LH group. Our series showed that the morbidity of AH was 6.4% (3/47), compared with 47.5% (28/59) in LH. To minimize surgical stress and operative risk only, nonanatomical limited hepatectomy should be a basic surgical procedure for RH. When taken surgical complications into account, AH is a safe and effective treatment, with a high stone clearance rate and fair rate of surgical complications. And AH should be the most effective treatment for hepatolithiasis and removes not only all of the hepatic stones but also the associated pathological bile ducts including stricture, fibrosis, abscess, and carcinomatous bile ducts [24]. 

For hepatolithiasis limited to a lobe (segment), irrespective of lobular atrophy or fibrosis, may receive AH, which results in radical cure and minimal affection on hepatic function; the stone located in several adjacent hepatic segments is also occurred frequently and may theoretically be cured by means of AH through complete removal of the stones, biliary strictures, and areas of postobstructive biliary dilation to prevent progressive liver damage and potential malignancy [8, 25]. However, not many patients received thorough resection of diseased liver tissues containing stones for the reason of protecting functioning hepatic cells as possible. The optimal management of these complex hepatolithiasis remains a very difficult and challenging task.

Our study can be criticized for its patient and treatment selection, the lack of assessment of quality-of-life issues when comparing AH and LH, as well as the retrospective nature of assessment of outcome. However, it highlights the possibility that AH does have a role in first-line management for RH. It can result in better clinical outcomes than LH with comparable morbidity and mortality. This forms the rationale for a randomized trial comparing AH with LH to circumvent the selection bias and address this controversial issue.

## Figures and Tables

**Table 1:** Characteristics of patients with regional hepatolithiasis treated by limited hepatectomy (LH) or anatomical hepatectomy (AH).

Variable	LH Group (*N* = 59)	AH Group (*N* = 47)	*P* Value
Sex (male/female)	21/38	14/33	.528
Age (year)	57.7 ± 1.2	59 ± 0.8	.453

Comorbidities			
Ischemic heart disease	9	7	.959
Diabetes	11	6	.413
Chronic obstructive airway disease	3	1	.779
Cirrhosis	5	2	.635

Laboratory test			
Serum bilirubin (umol/L)	34.3 ± 9.1	43.6 ± 4.7	.188
Serum albumin (g/L)	41.3 ± 2.6	42.0 ± 1.9	.403
Alanine transaminase (U/L)	80.7 ± 11.3	93.4 ± 9.8	.805
Aspartate transaminase (U/L)	77.8 ± 13.0	101.3 ± 3.7	.739
Alkaline phosphatase (U/L)	189.9 ± 26.2	217.2 ± 13.9	.225
r-Glutamyl transferase (U/L)	139.4 ± 11.2	151.8 ± 10.7	.442
Hemoglobin (g/dL)	11.8 ± 0.9	11.7 ± 1.6	.558
Platelet count (×10^9^/L)	271 ± 13.0	275 ± 17.6	.904
White blood cell count (×10^9^/L)	10.4 ± 0.4	10.9 ± 0.8	.670
Neutrophil (%)	79 ± 9.2	77 ± 8.6	.790
Prothrombin time (s)	14.3 ± 0.6	13.6 ± 1.3	.931
APTT (s)	31.8 ± 1.4	31.9 ± 1.8	.392
Serum hepatitis B surface antigen (positive/negative)	11/48	8/39	1.000
Indocyanine green clearance (%)	9.3 ± 0.9	8.3 ± 1.7	.172
Glucose (mmol/L)	7.4 ± 1.4	8.2 ± 0.9	.724

**Table 2:** Characteristics of regional hepatolithiasis treated by limited hepatectomy (LH) or anatomical hepatectomy (AH).

	LH (*N* = 59)	AH (*N* = 47)	*P* Value
Location of stones			
Left lobe	42	34	.896
Right lobe	9	7	.115
Bilateral lobe	8	6	.905

**Table 3:** Operative procedures for anatomical liver resection.

Location of stones		Number of patient	Surgical procedures
Left lobe (*n* = 34)	Segment II	7	Left lateral sectionectomy
	Segment III	11	Left lateral sectionectomy
	Segment II and III	12	Left lateral sectionectomy
	Segment IVa	1	S4a resection
	Segment II, III and IV	3	Left hemihepatectomy

Right lobe (*n* = 7)	Segment VI	4	S6 resection
	Segment VII	1	Right posterior sectionectomy
	Segment VI, and VII	1	Right posterior sectionectomy
	Segment V, VI, and VII	1	Right hemihepatectomy

Bilateral lobe (*n* = 7)	Segment II, III and VI	3	Left lateral sectionectomy + S6 resection
	Segment II, III and VII	2	Left lateral and right posterior sectionectomy
	Segment II, III and V, VIII	1	Left trisectionectomy

**Table 4:** Clinical Outcomes of patients treated by limited hepatectomy (LH) or anatomical hepatectomy (AH).

	LH (*N* = 59)	AH (*N* = 47)	*P* Value
Time of liver function recovery (d)	2.82 ± 0.131	2.96 ± 0.164	.745
Residual stone	13	2	.009*
Infection of the raw surface of liver remnant	21	1	.000*
Bile leakage	7	0	.040*
Recurrent stone	12	0	.001*
30-d mortality	0	0	
Total NO. of complications	52	3	.000*
Total NO. of patients with complications	28	3	.000*

*Shows statistical significance.
